# Cyclic Tests of Smooth and Notched Specimens Subjected to Bending and Torsion Taking into Account the Effect of Mean Stress

**DOI:** 10.3390/ma13092141

**Published:** 2020-05-06

**Authors:** Roland Pawliczek, Dariusz Rozumek

**Affiliations:** Department of Mechanics and Machine Design, Opole University of Technology, 45-271 Opole, Poland; d.rozumek@po.edu.pl

**Keywords:** mean stress, fatigue life, multiaxial stress, bending, torsion

## Abstract

The paper contains the results of fatigue tests of smooth and notched specimens made of 10HNAP (S355J2G1W) subjected to proportional cyclic loading with use of mean values stress. The results obtained for specimens under bending, torsion and one combination of bending with torsion for four mean values have been compared. The experimental data have been collected in the tables and shown in the figures with use of various σ_a_(τ_a_)-N fatigue characteristics for which parameters of the regression equations have been determined. The influence of average values on the allowable stress amplitudes and amplitude of moments at the level close to the fatigue limit depending on the angle α determining loading combination and the average stress is also shown. The greatest effect of the notch on fatigue life compared to smooth specimens is observed at symmetrical loads. At unsymmetrical loads with non-zero mean stress, this effect clearly weakens or disappears.

## 1. Introduction

Some structures are subjected to bending, torsion or a combination of both loads, which cause fatigue processes in materials. In addition, mean stresses occur in the structure load, resulting e.g., from the self-weight of the structure or the initial loads associated with the functions fulfilled by the structure. It is then necessary to adopt certain calculation models for fatigue life, taking into account the multiaxial load status and the effect of mean load values, and to perform their experimental verification [1,2,3,4,5]. In real structures, stress concentrations often occur due to the notch in the form of changes in cross-sectional dimensions, which significantly affect the fatigue strength of materials. Therefore, it becomes important to examine the impact of the notch on the fatigue life of the structure [6,7]. Modern methods of assessing fatigue life of machine elements can be divided into two groups. The first is characterized by an analytical approach based on spectral analysis of stochastic processes, while the second is characterized by an algorithmic approach based on numerical methods. These methods are constantly modified and improved so that you can meet the requirements currently set by designers.

Smaga et al. [8] present description on fatigue and transformation behavior at surrounding temperature and 300 °C of austenitic stainless steel AISI 347 in the whole fatigue range from Low Cycle Fatigue (LCF) to Very High Cycle Fatigue (VHCF). The test results show a significant effect of the chemical composition and temperature of induced martensite creation and the behavior of cyclic strain.

The local approach to predict the fatigue strength of sharply notched components can be applied to welded joints in a simplified form oriented to practical applications [9]. In this paper, satisfactory calculation results, based on the analytic expressions of the local stress field have been shown.

McDiarmid introduced some modifications of criterion determining the shear stress amplitude to take into account the stress mean value and the impact of the stress concentrator on the final result [10].

Rozumek et al. [11] gave a comparison between the experimental and calculated fatigue lives of specimens made of 10HNAP steel under non-proportional random bending with torsion including changes of strain energy density in the critical plane.

Literature sources present various review papers describing the characteristics of materials [12], which allow estimating the durability of structures and devices [13]. The ASTM [14] standards, which define and describe the method of determining stress (σ-N) and strain (ε-N) characteristics, are one of the most commonly used in the description of tests.

The purpose of the research described in the work is to investigate the behavior of smooth and notched specimens made of 10HNAP (S355J2G1W) steel under conditions of cyclic loading with the presence of various mean values. A wide database of material parameters describing its response for fatigue loads is necessary for the formulation and verification of strength calculation models. This data requires research and improvement for notched structures exposed to multiaxial fatigue loads.

## 2. Materials and Fatigue Test Stand

The subject of the study is 10HNAP (S355J2G1W) constructional steel included in the European Standard (EN 10155) “Low-alloy rust-resistant steel”. It is a general purpose weldable steel, with increased resistance to atmospheric corrosion. The content of elements in the tested steel is included in Table 1, and strength properties in Table 2.

**Figure 1:**
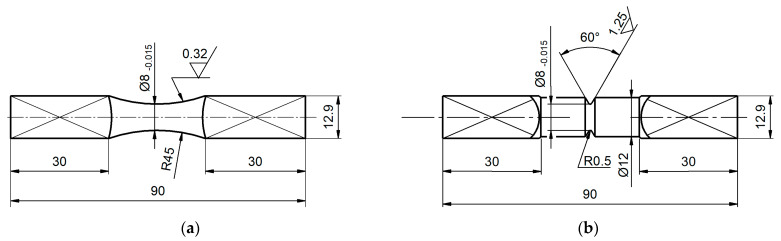
Shapes and dimensions of specimens: (**a**) smooth, (**b**) notched.

The specimens were cut from 16 mm thick sheet in accordance with the rolling direction. Figure 1 shows the shape and dimensions of the specimens. For a notched specimen the theoretical stress concentration factor, determined in accordance with Neuber’s model [15], is for bending K_tB_ = 2.46 and for torsion K_tT_ = 1.76.

The tests were carried out on the fatigue test stand MZGS-100 [16,17,18]. The stand used in the experiment allows testing for fatigue bending, torsion and proportional bending with torsion. The tests of cyclic bending and torsion were performed in low cycle fatigue (where number of cycles to failure is 10^4^–10^5^) and high cycle fatigue (where number of cycles to failure is 10^5^–10^7^) regime [19] with controlled load (in the considered case, the moment amplitude was controlled). The loads were of a sinusoidal nature under a frequency between 25–29 Hz. The amplitudes and the mean value were changed in accordance with the applied load. The nominal stress amplitude and nominal mean stress value (stress ratio) were used in calculations.

**Figure 2:**
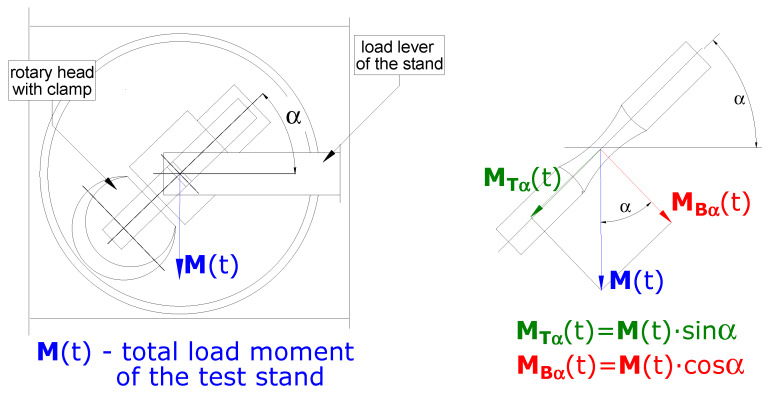
Load scheme of the specimen.

The load state of the specimen depends on the angular position of the rotary head of the stand, which is defined by the angle α determining the combination of bending and torsion (Figure 2).

The specimen load consists of a cyclically variable moment with amplitude *M_a_* and its mean value *M_m_*:

(1) $$M(t)=M_{m}+M_{a}sin\omega t,$$

where ω is the frequency of load changes.

The vectors of the torsional moment *M_T__α_(t)* and bending moment *M_B__α_(t)* (Figure 2) are related to the following relationships [20]

(2) $$M\left(t\right)=M_{T\alpha }(t)+M_{B\alpha }(t)$$

In the extreme position α = 0 rad the specimen is bent with a variable moment

(3) $$M_{B\alpha }(t)=M_{m}+M_{a}sin\omega t,$$

and in the position α = π/2 rad, the specimen is twisted with a torque

(4) $$M_{T\alpha }(t)=M_{m}+M_{a}sin\omega t,$$

The bending and torsional moments are related to the angle of rotation of the head with clamp (Figure 2) with proportion:

(5) $$\tan\alpha =\frac{M_{T\alpha }(t)}{M_{B\alpha }(t)}$$

In intermediate positions 0 < *α* < π/2 rad, both moments occur simultaneously and their values are determined from:

(6) $$M_{B\alpha }(t)=\left(M_{m}+M_{a}sin\omega t\right)cos\alpha ,\;\;\;\;M_{T\alpha }(t)=\left(M_{m}+M_{a}sin\omega t\right)sin\alpha ,$$

The result of the operation of both synchronized moments is a state of stress in which nominal normal stresses *σ_α_(t)* and shear stresses *τ_α_(t)* change their value according to phase and with the same angular frequency (*ω* = 29 Hz):

(7) $$\sigma_{\alpha }(t)=\sigma_{m\alpha }+\sigma_{a\alpha }sin\omega t,\;\;\;\;\tau_{\alpha }(t)=\tau_{m\alpha }+\tau_{a\alpha }sin\omega t,$$

where:

(8) $$\sigma_{\alpha }\left(t\right)=\frac{M_{B\alpha }\left(t\right)}{W_{x}},\;\;\;\;\tau_{\alpha }\left(t\right)=\frac{M_{T\alpha }\left(t\right)}{W_{0}}=\frac{M_{B\alpha }\left(t\right)}{2W_{x}},$$

*W_x_* and *W_0_* are section modulus for bending and torsion, respectively.

A stress ratio for bending *R_B_* and torsion *R_T_* are equal [21], since:

(9) $$R_{B}=\frac{\sigma_{min}}{\sigma_{max}}=\frac{\sigma_{a}-\sigma_{m}}{\sigma_{a}+\sigma_{m}}=\frac{\frac{M_{a}\cdot cos\alpha }{W_{x}}-\frac{M_{m}\cdot cos\alpha }{W_{x}}}{\frac{M_{a}\cdot cos\alpha }{W_{x}}+\frac{M_{m}\cdot cos\alpha }{W_{x}}}=\frac{\left(M_{a}-M_{m}\right)\frac{cos\alpha }{W_{x}}}{\left(M_{a}+M_{m}\right)\frac{cos\alpha }{W_{x}}}=\frac{\left(M_{a}-M_{m}\right)}{\left(M_{a}+M_{m}\right)},$$

and

(10) $$R_{T}=\frac{\tau_{min}}{\tau_{max}}=\frac{\tau_{a}-\tau_{m}}{\tau_{a}+\tau_{m}}=\frac{\frac{M_{a}\cdot sin\alpha }{W_{0}}-\frac{M_{m}\cdot sin\alpha }{W_{0}}}{\frac{M_{a}\cdot sin\alpha }{W_{0}}+\frac{M_{m}\cdot sin\alpha }{W_{0}}}=\frac{\left(M_{a}-M_{m}\right)\frac{sin\alpha }{W_{0}}}{\left(M_{a}+M_{m}\right)\frac{sin\alpha }{W_{0}}}=\frac{\left(M_{a}-M_{m}\right)}{\left(M_{a}+M_{m}\right)},$$

so *R_B_ = R_T_ = R*.

Main stresses in the plane stress state considered here on the surface of the specimen (*σ_1_* ≠ 0, *σ_3_* ≠ 0 and *σ_2_* = 0) can be saved for the condition *R_B_ = R_T_* and compatible phases as

(11) $$\sigma_{1\alpha }(t)=\sigma_{1m\alpha }+\sigma_{1a\alpha }sin\omega t,\;\;\;\;\sigma_{3\alpha }(t)=\sigma_{3m\alpha }+\sigma_{3a\alpha }sin\omega t,$$

where:

(12) $$\sigma_{1,3m\alpha }=\frac{1}{2}\left(\sigma_{m\alpha }\pm \sqrt{\sigma_{m\alpha }^{2}+4\tau_{m\alpha }^{2}}\right),\;\;\;\;\sigma_{1,3a\alpha }=\frac{1}{2}\left(\sigma_{a\alpha }\pm \sqrt{\sigma_{a\alpha }^{2}+4\tau_{a\alpha }^{2}}\right),$$

Directions of main stresses in relation to stress *σ_α_ (t)* are rotated by angles

(13) $$\Psi_{1}=\frac{1}{2}arctg\frac{-2\tau_{\alpha }(t)}{\sigma_{\alpha }(t)},\;\Psi_{3}=\Psi_{1}+\frac{\pi }{2}$$

## 3. The Terms of Fatigue Tests

Smooth and notched specimens made of 10HNAP (S355J2G1W) steel were subjected to fatigue testing. Fatigue tests included synchronous, sinusoidal variable loads with an additional mean loads. The tests covered three states of specimens loading. The following mean stresses were used for individual values of the angle α of rotation of the machine head

(a) *α* = 0 rad (bending),
  *σ_m_* = 0 MPa,
  *σ_m_* = 75 MPa,
  *σ_m_* = 150 MPa,
  *σ_m_* = 225 MPa.
(b) *α* = 1.107 rad (63.5°) (bending with torsion) $M_{g\alpha }\left(t\right)=2M_{s\alpha }\left(t\right)\;;\;\sigma_{\alpha }\left(t\right)=\tau_{\alpha }\left(t\right)$
  *σ_m_* = *τ_m_* = 0 MPa,
  *σ_m_* = *τ_m_* = 36 MPa,
  *σ_m_* = *τ_m_* = 75 MPa,
  *σ_m_* = *τ_m_* = 114 MPa.
(c) *α* = π/2 rad (torsion)
  *τ_m_* = 0 MPa,
  *τ_m_* = 75 MPa,
  *τ_m_* = 150 MPa,
  *τ_m_* = 225 MPa.

The tests were performed for 4–5 levels of stress amplitude, 2–3 specimens at each load level.

## 4. Fatigue Characteristics of the Material

The results of fatigue tests were approximated by the following regression Equations [14]:

(14) $$logN=A+mlog\sigma_{a}$$

(15) $$logN=A+mlog\tau_{a}$$

where: *σ_a_*—bending stress amplitude, *τ_a_*—torsional stress amplitude, *N*—number of cycles for failure, *A* and *m*—parameters of the regression equation.

Table 3 and Table 4 contain the numerical values of the parameters of the regression Equations (14) and (15), as well as the values of the correlation coefficient r for variables *logN* and *logσ_a_* and logarithm variance values from the number of cycles *N*, i.e., $\mu_{logN}$ for smooth and notched specimens at various load combinations. The parameter *m*, which in the double-logarithmic coordinate system is the slope coefficient *σ_a_*—*N*, takes negative values and shows fluctuations. The largest changes are observed in the case of bending, where m is clearly smaller for smooth specimens compared to notched specimens. For torsion there is no such clear tendency and differences between *m* coefficient are not so strong as in bending case. The opposite trend to bending occurs for combining bending with torsion where mean stress value is greater than 0. Negative values of the correlation coefficient *r* in both tables indicate a strong relationship between the logarithm of the number of cycles to failure and the logarithm of the stress amplitude for smooth and notched specimens at all types of loads.

**Figure 3:**
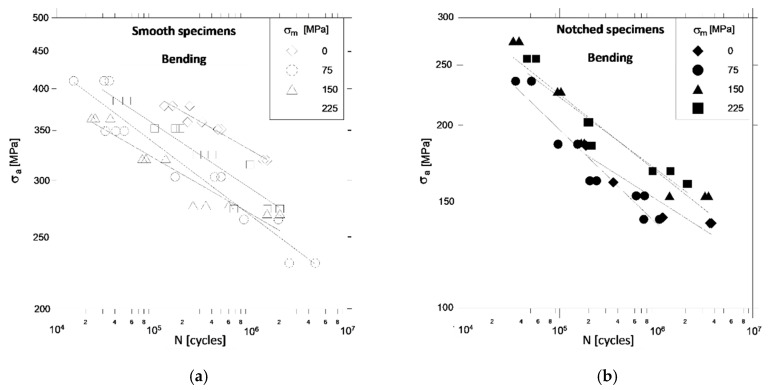
Comparison of fatigue characteristics of smooth (**a**) and notched (**b**) specimens under bending and different values of mean stress.

**Figure 4:**
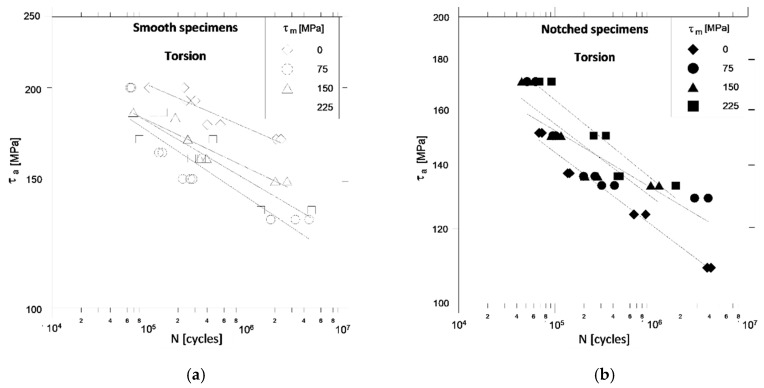
Comparison of fatigue characteristics of smooth (**a**) and notched (**b**) specimens under torsion and different values of mean stress.

**Figure 5:**
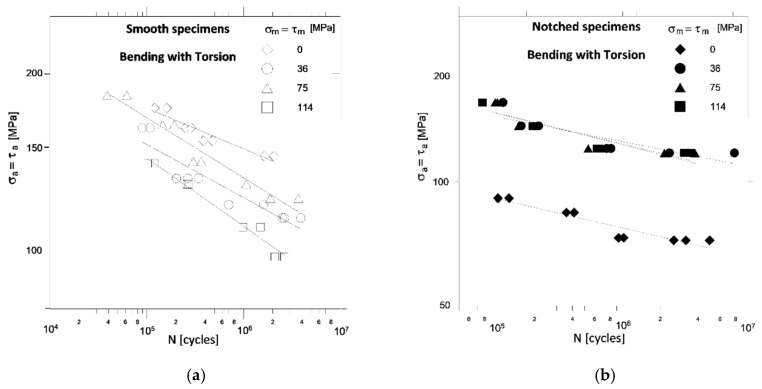
Comparison of fatigue characteristics of smooth (**a**) and notched (**b**) specimens under combined bending with torsion and different values of mean stress.

Figure 3, Figure 4 and Figure 5 show the fatigue characteristics of specimens for bending, torsion and the combination of bending and torsion, to illustrate the impact of notch and mean stress value on fatigue life.

Figure 3 shows that with pure bending, the presence of a notch in the specimens significantly reduces the allowable stress amplitudes in relation to smooth specimens for the same fatigue life.

The increase in mean stresses *σ_m_* when bending smooth samples (Figure 3a) in the range of 0–150 MPa reduces (as expected) the allowable stress amplitudes *σ_a_*, while at *σ_m_* = 225 MPa it increases unexpectedly *σ_a_*. The growth trend *σ_a_*, with the increase *σ_m_*, for a constant number of cycles to damage, is more clearly manifested in notched specimens (Figure 3b). These observations lead to the conclusion that from certain, sufficiently high levels of mean stress in smooth specimens and from much lower levels *σ_m_* notched specimens have large plastic strains and cyclic strengthening of the material.

Taking into account the Neuber’s model with specified factor *K_tB_* = 2.46 and using Goodman model for mean stress correction it was found, that for the case of smooth specimens and *σ_m_* = 225 MPa maximum stress varies from 455 MPa for HCF up to 640 MPa for LCF range. These stresses exceed the yield stress of material. Meanwhile, for notched specimens, these stresses are respectively 512 MPa and 920 MPa for *σ_m_* = 150 MPa and 650 MPa and 150 MPa for *σ_m_* = 225 MPa.

Additional factors that should be taken into account when explaining these observations are the presence of biaxial stress states in the notch bottom and stress gradients in the cross sections of the specimens. With pure torsion, the presence of a notch in the samples weakens them compared to smooth specimens (Figure 4), but to a lesser extent than is observed when bending (Figure 3).

The influence of mean shear stresses *τ_m_* on the permissible amplitudes of shear stresses *τ_a_* for smooth specimens (Figure 4a) and notched specimens (Figure 4b) with constant life is similar here as for bending. When comparing the fatigue diagrams of smooth specimens (Figure 3a and Figure 4a) and notched specimens (Figure 3b and Figure 4b) under bending and torsion, it can be seen that the mean stresses *σ_m_* and *τ_m_* strongly affect the allowable stress amplitudes *σ_a_* and *τ_a_* in smooth specimens than in notched specimens, where in turn the effects that can be the result of cyclic strengthening of the material are more pronounced. Manifested in an increase in allowable stress amplitudes with an increase in mean stresses for the same number of cycles for damage. With the combination of bending and torsion (Figure 5), the strongly weakening effect of the specimens notch is observed only with symmetrical cycles (*σ_m_ =* *τ_m_* = 0), while with asymmetrical cycles (*σ_m_ =* *τ_m_* > 0) this effect is blurred and can be assume that it disappears. Increasing mean stresses *σ_m_ =* *τ_m_* for smooth specimens (Figure 5a) basically reduces the allowable stress amplitudes *σ_a_ =* *τ_a_*, while for notched specimens (Figure 5b) it unexpectedly increases the allowable stress amplitudes. To determine the equivalent stress for a complex load, the Huber-Mises hypothesis was used. The effect of the mean load value was taken into account with the Goodman correction and with the use of the Neuber’s model notch effect was included (*K_tB_* = 2.46, *K_tT_* = 1.76). For smooth specimens the maximum stress varies from 260 MPa for HCF up to 500 MPa for LCF regime. In the case of notched specimens (Figure 5b), these stresses exceed the yield stress of material and they are 660 MPa and 873 MPa for *σ_m_* = *τ_m_* = 36 MPa, 783 MPa, 1037 MPa for *σ_m_* = *τ_m_* = 75 MPa, 964 MPa and 1227 MPa for *σ_m_* = *τ_m_* = 114 MPa.

Table 5 shows values of the fatigue limits for the material. In the case of bending with torsion, providing the fatigue limit value requires the adoption of an appropriate multiaxial fatigue criterion, which will be the subject of analysis in a separate work.

**Figure 6:**
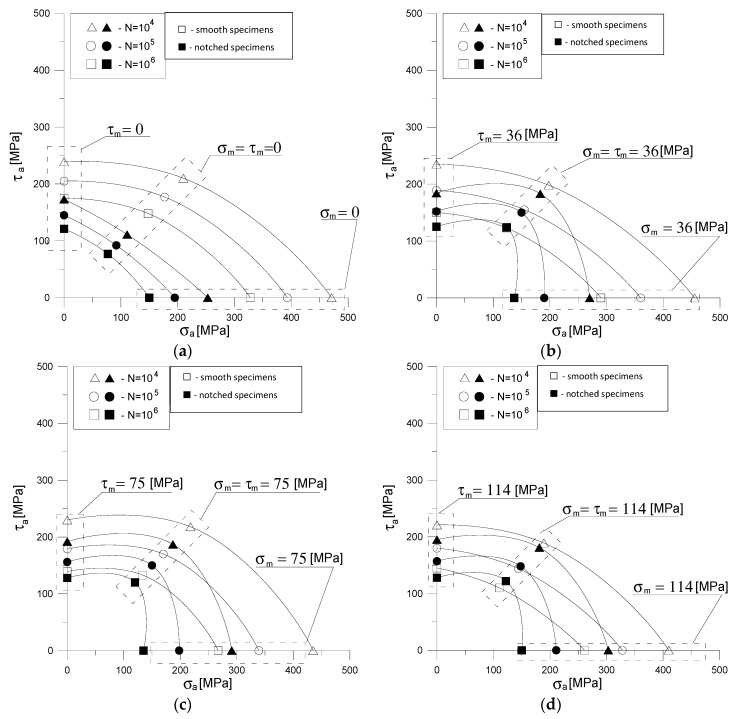
Graphs of bending and torsion stress of smooth and notched specimens for various fatigue life and (**a**) *σ_m_ =* *τ_m_* = 0, (**b**) *σ_m_ =* *τ_m_* = 36, (**c**) *σ_m_ =* *τ_m_* = 75, (**d**) *σ_m_ =* *τ_m_* = 114 MPa.

Based on the parameters of the regression equations in Table 3 and Table 4, charts were drawn for the impact of mean values *σ_m_ =* *τ_m_* = 0, 36, 75 and 114 MPa on the allowable stress amplitudes for the three selected number of cycles for damage N = 10^4^, 10^5^ and 10^6^ smooth and notched specimens (Figure 6). Lines approximating the results of Figure 6 obtained by interpolation. Thanks to these graphs, forecasting of fatigue life or allowable stress amplitudes for other load combinations than *α* = 1.107 rad becomes possible without conducting additional tests. Figure 6a shows that with symmetrical load combinations, the presence of a notch on the specimens reduces the allowable stress amplitudes by about 50% compared to the stress amplitudes for smooth specimens at the same lifetime. However, in Figure 6b–d it can be seen that the impact of the notch on the permissible stress amplitudes decreases when non-zero mean stresses appear. In Figure 6d an unexpectedly favorable effect of the notch is observed at more cycles for damage (10^5^; 10^6^ cycles) for the combination of bending and torsion with the mean stress *σ_m_ =* *τ_m_* = 114 MPa.

**Figure 7:**
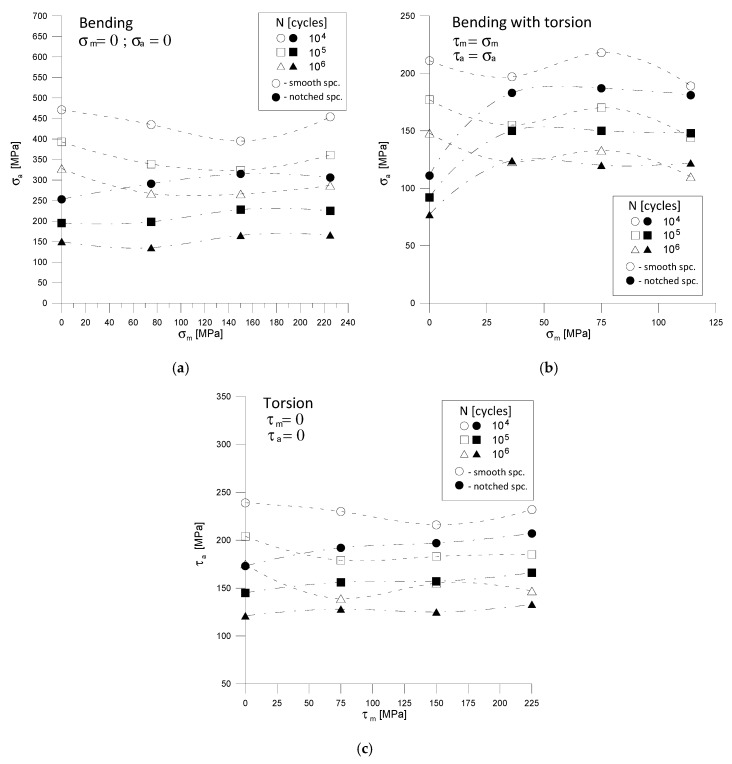
Influence of mean stress values on permissible stress amplitudes: (**a**) for bending, (**b**) for bending with torsion, (**c**) for torsion.

The parameters of the regression equations from Table 3 and Table 4 were also used to illustrate in Figure 7 the impact of mean stresses *σ_m_* and *τ_m_* on the permissible stress amplitudes *σ_a_* and *τ_a_* for three different combinations of loads and three fatigue life *N* = 10^4^, 10^5^ and 10^6^ cycles.

In the graphs (Figure 7) there is a lack of a clear decrease in the stress amplitudes *σ_a_* and *τ_a_* with an increase in mean stresses, respectively, *σ_m_* and *τ_m_* as would be expected according to many well-known graphs, e.g., Haigh, Soderberg [21], for smooth specimens. In the case of notched specimens is surprising increase in the stress amplitudes *σ_a_* and *τ_a_* with the increase in mean stresses *σ_m_* and *τ_m_*, which with pure bending and pure torsion is insignificant and explicit with the combination of bending and torsion.

**Figure 8:**
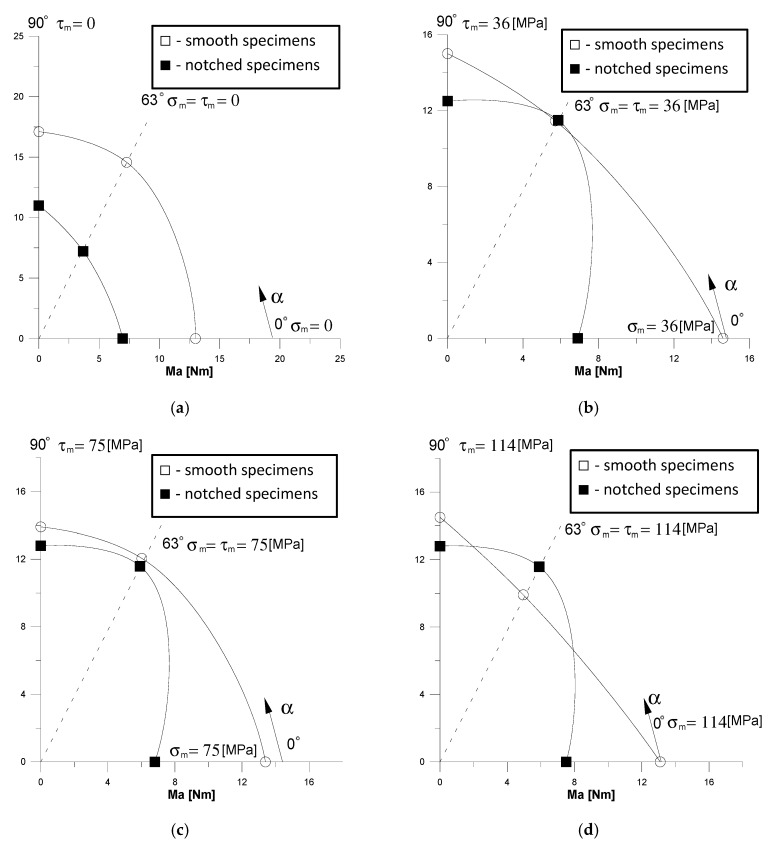
Amplitudes of moments at the level close to the fatigue limit (N = 10^6^ cycles) depending on angle α and mean stress level: (**a**) 0, (**b**) 75 MPa, (**c**) 150 MPa, (**d**) 225 MPa.

Permissible values of amplitudes of the total moment of the loading force at a level close to the fatigue limit (*N* = 10^6^ cycles) depending on the type of nominal stresses (*α* = 0, 63° and 90°), as well as the values of mean stress (*σ_m_,* *τ_m_*) and notch shown in Figure 8. Figure 8 shows that the lowest values of moment amplitudes are required to damage notched specimens under bending and they are on average twice as large as the moment amplitudes for damage of these specimens when torsion. Such significant differences in the values of amplitudes for pure bending and pure torsion are not observed in the case of smooth specimens. At asymmetrical loads of notched specimens, the levels of torque amplitudes under pure torsion and the tested combination of bending and torsion are similar (Figure 8b–d). Figure 8d shows again that with the combination of bending and torsion larger moments are needed to damage notched specimens than smooth specimens. This anomaly deserves a separate, elastic-plastic analysis of the phenomena observed in specimens from the tested material.

The effect of negligible small influence of mean shear stress on fatigue life, especially for high cycle fatigue regime, is well known [21,22]. The mean stress value can involve the ratcheting effect in material and phenomena of ratcheting strains can influence on damage accumulation process.

Additionally, investigation of 10HNAP (S355J2G1W) steel shows that for fatigue limit the coefficient of the cycles asymmetry sensitivity for torsion is about two times smaller in comparison to bending loads [23]. Assuming a negligible effect of the shear means stress the allowable amplitude of the loads at the fatigue limit.

## 5. Conclusions and Finding

The following conclusions can be drawn from the tests of smooth and notched specimens made of 10HNAP steel at cyclic loads, taking into account the effect of mean stress values

1 Notch significantly reduces the allowable nominal stress amplitudes in the case of pure bending and pure torsion, while the effect is smaller in the combination of bending and torsion. These observations confirm the typical behavior of structural steels in machine elements where stress concentrations occur.
2 The greatest effect of the notch on fatigue strength compared to smooth specimens is observed at symmetrical loads. At unsymmetrical loads with non-zero values of mean stress, this effect clearly weakens or disappears and incidentally turned out to be unexpectedly opposite to the general trend in a large number of cycles.
3 In smooth specimens with sufficiently high mean stresses (*σ_m_ =* *τ_m_* = 225 MPa) and for significantly lower mean stress levels (*σ_m_ =* *τ_m_* = 36 MPa) in notched specimens there appears the effect of strengthening the material, which indicates the presence of significant plastic deformations.
4 The calculation models proposed for assessing the fatigue life of 10HNAP steel under bending, torsion and combination of bending with torsion taking into account the value of mean stresses and the presence of notches should take into account the appearance of significant local plastic strains, cyclic strengthening of the material and the occurrence of stress gradients in the cross-sections of the rods. Proposals for such models will be the subject of a separate publication by the authors.

## Figures and Tables

**Table 1:** Chemical composition of 10HNAP steel (wt %).

C-0.115	Mn-0.52	Si-0.26	P-0.098
S-0.016	Cr-0.65	Ni-0.35	Cu-0.26

**Table 2:** Mechanical properties of 10HNAP steel.

Yeld Strength σ_Y_, MPa	Ultimate Strength σ_U_, MPa	Young Modulus E, GPa	Poisson’s Ratio ν
418	566	215	0.29

**Table 3:** Parameters of regression equations describing fatigue life of smooth specimens.

Parameters of Regression Equations	Bending		Bending with Torsion		Torsion	
	*α* = 0°		*α* = 63.5° (*τ_a_* *=* *σ_a_*) (*τ_m_* *=* *σ_m_*)		*α* = 90°	
*A*	σ_m_ = 0	38.03	σ_m_ = 0	34.62	τ_m_ = 0	39.28
	σ_m_ = 75	28.41	σ_m_ = 36	26.17	τ_m_ = 75	25.50
	σ_m_ = 150	34.30	σ_m_ = 75	25.63	τ_m_ = 150	36.45
	σ_m_ = 225	30.78	σ_m_ = 114	23.31	τ_m_ = 225	28.00
*m*	σ_m_ = 0	−12.73	σ_m_ = 0	−13.18	τ_m_ = 0	−14.83
	σ_m_ = 75	−9.25	σ_m_ = 36	−9.66	τ_m_ = 75	−9.10
	σ_m_ = 150	−11.67	σ_m_ = 75	−9.25	τ_m_ = 150	−13.90
	σ_m_ = 225	−10.08	σ_m_ = 114	−8.48	τ_m_ = 225	−10.15
correlation coefficient r	σ_m_ = 0	−0.97	σ_m_ = 0	−0.95	τ_m_ = 0	−0.92
	σ_m_ = 75	−0.96	σ_m_ = 36	−0.93	τ_m_ = 75	−0.90
	σ_m_ = 150	−0.94	σ_m_ = 75	−0.97	τ_m_ = 150	−0.94
	σ_m_ = 225	−0.94	σ_m_ = 114	−0.99	τ_m_ = 225	−0.89
variance *μ_logN_*	σ_m_ = 0	9.30 × 10^−3^	σ_m_ = 0	2.13 × 10^−2^	τ_m_ = 0	3.81 × 10^−2^
	σ_m_ = 75	6.07 × 10^−2^	σ_m_ = 36	5.34 × 10^−2^	τ_m_ = 75	1.01 × 10^−1^
	σ_m_ = 150	5.50 × 10^−2^	σ_m_ = 75	2.86 × 10^−2^	τ_m_ = 150	3.90 × 10^−2^
	σ_m_ = 225	4.56 × 10^−2^	σ_m_ = 114	8.22 × 10^−3^	τ_m_ = 225	8.67 × 10^−2^

**Table 4:** Parameters of regression equations describing fatigue life of notched specimens.

Parameters of Regression Equations	Bending		Bending with Torsion		Torsion	
	*α* = 0°		*α* = 63.5° (*τ_a_* *=* *σ_a_*) (*τ_m_* *=* *σ_m_*)		*α* = 90°	
*A*	σ_m_ = 0	25.13	σ_m_ = 0	29.84	τ_m_ = 0	32.74
	σ_m_ = 75	18.73	σ_m_ = 36	30.45	τ_m_ = 75	29.84
	σ_m_ = 150	21.81	σ_m_ = 75	27.74	τ_m_ = 150	27.26
	σ_m_ = 225	22.72	σ_m_ = 114	30.34	τ_m_ = 225	28.2
*m*	σ_m_ = 0	−8.79	σ_m_ = 0	−12.64	τ_m_ = 0	−12.84
	σ_m_ = 75	−5.98	σ_m_ = 36	−11.69	τ_m_ = 75	−11.32
	σ_m_ = 150	−7.13	σ_m_ = 75	−10.45	τ_m_ = 150	−10.14
	σ_m_ = 225	−7.53	σ_m_ = 114	−11.67	τ_m_ = 225	−10.45
correlation coefficient r	σ_m_ = 0	−0.94	σ_m_ = 0	−0.94	τ_m_ = 0	−0.99
	σ_m_ = 75	−0.96	σ_m_ = 36	−0.89	τ_m_ = 75	−0.83
	σ_m_ = 150	−0.95	σ_m_ = 75	−0.90	τ_m_ = 150	−0.88
	σ_m_ = 225	−0.94	σ_m_ = 114	−0.93	τ_m_ = 225	−0.93
variance μ_logN_	σ_m_ = 0	3.64 × 10^−2^	σ_m_ = 0	4.67 × 10^−2^	τ_m_ = 0	1.29 × 10^−2^
	σ_m_ = 75	2.48 × 10^−2^	σ_m_ = 36	1.18 × 10^−1^	τ_m_ = 75	1.49 × 10^−1^
	σ_m_ = 150	7.06 × 10^−2^	σ_m_ = 75	8.38 × 10^−2^	τ_m_ = 150	7.27 × 10^−2^
	σ_m_ = 225	4.61 × 10^−2^	σ_m_ = 114	6.89 × 10^−2^	τ_m_ = 225	4.31 × 10^−2^

**Table 5:** Fatigue limits of smooth and notched specimens for different values of mean stress.

*σ_m_*, MPa	Smooth Specimens		Notched Specimens	
	Bending	Torsion	Bending	Torsion
0	298	169	137	109
75	229	130	137	124
150	266	138	161	129
225	261	134	161	129
